# Do elevated blood levels of omega-3 fatty acids modify effects of particulate air pollutants on fibrinogen?

**DOI:** 10.1007/s11869-018-0586-0

**Published:** 2018-06-03

**Authors:** Daniel Croft, Robert Block, Scott J. Cameron, Kristin Evans, Charles J. Lowenstein, Frederick Ling, Wojciech Zareba, Philip K. Hopke, Mark J. Utell, Sally W. Thurston, Kelly Thevenet-Morrison, David Q. Rich

**Affiliations:** 1 Division of Pulmonary and Critical Care, Department of Medicine, University of Rochester Medical Center, 601 Elmwood Avenue, Box 692, Rochester, NY 14642 USA; 2 Division of Cardiology, Department of Medicine, University of Rochester Medical Center, Rochester, NY USA; 3 Department of Public Health Sciences, University of Rochester Medical Center, Rochester, NY USA; 4 Institute for a Sustainable Environment, and Center for Air Resources Engineering and Science, Clarkson University, Potsdam, NY USA; 5 Department of Environmental Medicine, University of Rochester Medical Center, Rochester, NY USA; 6 Department of Biostatistics and Computational Biology, University of Rochester, Rochester, NY USA

**Keywords:** Omega-3 fatty acids, Fish oil, Fibrinogen, Particulate matter, Effect modification, Nutritional factors

## Abstract

Previously, we found short-term increases in ambient particulate matter (PM) air pollutant concentrations were associated with increased serum fibrinogen levels in patients with cardiac disease. We now studied whether high blood levels of omega-3 (ω-3) fatty acids blunted this fibrinogen response to increased PM concentrations in these same patients. Plasma fibrinogen and ω-3 fatty acid levels (% of total identified fatty acids) were measured in blood samples collected from 135 patients treated at the University of Rochester Medical Center for myocardial infarction or stable ischemic heart disease requiring cardiac catheterization. Using ambient measurements of ultrafine, accumulation mode, and fine particles (PM_2.5_), Delta-C, and black carbon (BC), we regressed serum fibrinogen levels against pollutant concentrations over the previous 1–96 h, using interaction terms to estimate these associations separately for those with HIGH (> 5.12%) and LOWMED serum levels of ω-3 fatty acid (≤ 5.12%). Each 5.6 μg/m^3^ increase in PM_2.5_ concentration in the previous hour was associated with a 3.1% increase in fibrinogen (95% CI = 1.5%, 4.7%) in those subjects with LOWMED total ω-3 fatty acid levels, but only a 0.9% increase (95% CI = − 1.5%, 3.2%) in patients with HIGH total ω-3 fatty acid levels. This same pattern was observed with fish oil-derived docosahexaenoic and eicosapentaenoic acids but not alpha-linolenic (from plant oil or seeds). A similar finding was observed with BC in the prior 24 h, but not other PM. Thus, increased blood levels of fish-based ω-3 fatty acids attenuated increases in fibrinogen associated with short-term increases in ambient PM.

**Electronic supplementary material:**

The online version of this article (10.1007/s11869-018-0586-0) contains supplementary material, which is available to authorized users.

## Introduction

Particulate air pollution (PM) has repeatedly been associated with an increased risk of acute cardiovascular events like myocardial infarction (Brook et al. 2010). Other studies have reported associations between short-term increases in ambient air pollutants and adverse changes in plasma biomarkers of inflammation and coagulation (Rich et al. 2012a, b; Ruckerl et al. 2007), which are potential mediators of any pollutant effect on these acute cardiovascular events. Specifically, the acute phase reactant fibrinogen has been associated with increases in PM concentrations at lag times of 24–72 h following exposure (Chuang et al. 2007; Rich et al. 2012b) with our prior study showing an association at lag times as short as 1 h (Croft et al. 2017). Biologically, fibrinogen has been observed to increase within 6 h after an inflammatory stimulus with a peak response typically observed at 96 h (Colley et al. 1983).

Previous studies have examined whether intake of omega 3 (ω-3) fatty acids can reduce the risk of acute cardiovascular events and overall risk of cardiovascular disease (Harris et al. 2008). ω-3 fatty acids include eicosapentaenoic acid (EPA) and docosahexaenoic acid (DHA), which are obtained from marine sources such as fish, and alpha-linolenic acid (ALA), a plant-derived fatty acid present in the oils of seeds, nuts, and beans (Harris et al. 2008). Docosapentaenoic acid (DPA) is an ω-3 fatty acid intermediary in the metabolism of EPA to DHA and is generally found in lower concentrations than either of these other fatty acids in foods, with the exception of human milk (Byelashov et al. 2015). Adequate DHA, EPA, and ALA blood concentrations have all been shown to be protective for cardiovascular disease (Chaddha and Eagle 2015; Mozaffarian and Wu 2011) including acute coronary syndrome (ACS) events (de Oliveira Otto et al. 2013). While the cardioprotective effects of ω-3 fatty acids remain an active area of research, the American Heart Association currently states that supplementation with DHA and EPA is reasonable for patients with recent MI or heart failure with reduced ejection fraction (Siscovick et al. 2017).

The cardioprotective effects of ω-3 fatty acids are thought to involve a multifactorial mechanism including anti-inflammatory (Chapkin et al. 2009) and anti-platelet effects (Abdolahi et al. 2014; Block et al. 2013; Block et al. 2015). Previous studies have shown that fish oil supplements (omega-3 fatty acids) blunted ambient air pollutant effects on measures of autonomic dysfunction (i.e., heart rate variability) (Romieu et al. 2005). Also, Tong et al. (2015) observed attenuation of increases in fibrinogen associated with PM in subjects taking fish or olive oil supplements. Our study examines whether specific components of ω-3 fatty acids (whether fish or plant based) can blunt acute ambient air pollutant impacts on fibrinogen in cardiac patients.

Previously, in a panel of patients treated for acute coronary syndrome or stable coronary artery disease in our cardiac catheterization laboratory, we found that increased ambient PM concentrations in the previous 1–48 h were associated with approximately 1–3% increases in plasma fibrinogen levels (Croft et al. 2017). Using these same fibrinogen, pollutant, clinical data, and ω-3 fatty acids measured in these same plasma samples, we hypothesized that in those subjects with lower ω-3 fatty acids levels, we would see increases in fibrinogen associated with acute increases in PM concentrations (1–96 h lags), but little to no increase in fibrinogen associated with increased PM in those subjects with high ω-3 fatty acids levels. We also hypothesized that the fish-based ω-3 fatty acids (EPA and DHA) would modify these same PM/fibrinogen associations, but not the plant-based ω-3 fatty acid (ALA).

## Methods

### Study population

The study population has been described previously (Croft et al. 2017). Briefly, we included adult (≥ 18 years of age) patients treated at the University of Rochester Medical Center (URMC) Cardiac Catheterization Laboratory, during the winters (November 1st to April 30th) of 2011, 2012, or 2013, for either ST-elevation myocardial infarction (STEMI; *n* = 25), non-ST elevation myocardial infarction (NSTEMI; *n* = 32), or stable ischemic heart disease requiring cardiac catheterization (SIHD) (*n* = 78). After informed consent was received, we atraumatically collected a 30-mL blood sample at the time of cardiac catheterization from each patient.

### Biomarkers

Plasma concentrations of fibrinogen (μg/ml) were measured using an enzyme-linked immunoassay (ELISA). The plasma phospholipid fatty acid composition of ω-3 fatty acids (EPA, DHA, ALA, and DPA) was analyzed by gas chromatography (GC) with flame ionization detection, which has similar testing characteristics as the standard thin layer chromatography method (Harris et al. 2007). Plasma concentrations of ω-3 fatty acids were reported as a percent of total identified fatty acids. All study activities were approved by the University of Rochester Research Subjects Review Board.

### Air pollution and weather data

Full details of our ambient air pollutant measurement methods have been described previously (Croft et al. 2017). Briefly, hourly pollutant measurements were made at the New York State Department of Environmental Conservation (NYSDEC) site in Rochester, NY, and averaged to hourly concentrations. Particulate matter (PM_2.5_) was measured continuously using a tapered element oscillating microbalance (TEOM; model 1400ab, ThermoFisher, Franklin, MA). Black carbon (BC) (marker of traffic pollution) was measured using a two-wavelength (370 and 880 nm) aethalometer (Magee Scientific, Inc., Berkeley, CA) with BC measured using the 880-nm wavelength and Delta-C (a marker of wood smoke) calculated as the difference between the BC measured at 370 and 880 nm (Wang et al. 2012). AMP (100–500 nm diameter) and UFP (10–100 nm diameter) were both measured using a 3071 Electrostatic Classifier with a 3010 Condensation Particle Counter (TSI Inc., St. Paul, MN) functioning as a scanning mobility particle sizer (SMPS). Ambient temperature and relative humidity were continuously measured at the same NYSDEC site.

### Statistical analysis

For our primary analysis, all pollutant specific analyses described below used those lag times associated with the largest change in fibrinogen in our previous analyses (i.e., BC in the previous 24 h, Delta-C and AMP in the previous 12 h, PM_2.5_ in the previous 1 h, and UFP in the previous 48 h) (Croft et al. 2017). First, we separated all subjects into tertiles (LOW, MEDIUM, HIGH) based on their ω-3 fatty acids level. Second, using linear regression, we regressed fibrinogen against the mean PM_2.5_ concentration in the previous 1 h, including indicator variables for ω-3 tertile, interaction terms between ω-3 tertiles and PM_2.5_ (PM_2.5_*LOW, PM_2.5_*MEDIUM), and indicator variables for age (< 50, 50–59, 60–69, 70–79, > 79 years old), year (2011, 2012, or 2013), weekday (weekday versus weekend), hour of the day (04:00–11:59 versus 12:00–03:59), dyslipidemia, prior MI, and smoking (current versus not-current), as well as natural splines (3 degrees of freedom) of temperature (degrees Fahrenheit, prior 24 h), and relative humidity (%, prior 24 h). Except for the indicator variables for ω-3 tertile and the interaction terms, this was the same model used in our previous analyses (Croft et al. 2017). Third, we re-ran the models described above, but with only two ω-3 groups per analysis (i.e., LOW and MEDIUM tertiles combined [LOWMED] versus the HIGH tertile). As a secondary objective, we separately re-ran the same models to examine effect modification by each specific ω-3 fatty acid (DHA, EPA, ALA, and DPA). Finally as a sensitivity analysis, we re-ran the same model for each lagged PM_2.5_, BC, UFP, AMP, and Delta-C concentration (lag hours 0, 0–11, 0–23, 0–47, 0–71, and 0–95). All analyses were performed using SAS version 9.4 (SAS Institute Inc., Cary, NC) with statistical significance defined as *p* < 0.05.

## Results

The 135 patients in the study were primarily Caucasian (96%), male (73%), over 60 years of age (63%), and overweight or obese (83%), with cardiovascular comorbidities including hypertension (76%) and dyslipidemia (67%) (Table 1). The distributions of pollutant concentrations used in the analysis are shown in Table 2, the distribution of ω-3 fatty acids in Table 3, and the distribution of fibrinogen within ω-3 fatty acids tertiles in Table S1.

**Table 1:** Characteristics of study subjects (*N* = 135)

	*N* (%)^a^
Age	
30–59 years	51 (38)
60–69 years	44 (33)
70–89 years	40 (30)
Male	98 (73)
Race/ethnicity	
Non-Hispanic White	129 (96)
Non-Hispanic Black, Hispanic, other	6 (4)
Category of cardiac disease	
SIHD	78 (58)
NSTEMI	32 (24)
STEMI	25 (19)
Prior cardiovascular disease	42 (31)
Prior myocardial infarction	12 (9)
Prior percutaneous intervention	27 (20)
Prior coronary artery bypass	14 (10)
Prior peripheral artery disease	10 (7)
Prior heart failure	10 (7)
Smoker^b^	37 (28)
Hypertension	103 (76)
Dyslipidemia	90 (67)
Diabetes mellitus	34 (25)
Body mass index (BMI: kg/m^2^)	
Normal (BMI 18.5–24.9)	23 (17)
Overweight (BMI 25.0–29.9)	44 (33)
Obesity: class I (BMI 30.0–34.9)	40 (30)
Obesity: class II (BMI 35.0–39.9)	14 (10)
Obesity: class III (BMI > 40)	14 (10)

^a^Categories may not sum to 100% due to rounding

^b^1 subject had missing data on smoking

**Table 2:** Distribution of pollutant concentrations and weather characteristics at specific lag times in the winters of 2011–2013

Parameter	Lag hours	*N*	Mean	Standard deviation	25th	50th	75th	Interquartile range
Accumulation mode particles (AMP) (n/cm^3^)	0–11	135	507	367	225	417	677	452
Black carbon (μg/m^3^)	0–23	135	0.37	0.23	0.2	0.29	0.49	0.29
Delta-C (μg/m^3^)	0–11	135	0.14	0.21	0.03	0.08	0.17	0.13
PM_2.5_ (μg/m^3^)	0	127	6.8	3.4	4.1	6.1	9.7	5.6
Ultrafine particles (UFP) (n/cm^3^)	0–47	135	2880	1182	2033	2742	3628	1595

**Table 3:** Distribution of ω-3 fatty acids among study patients from 2011 to 2013

ω-3 (% of total fatty acids)	*N*	Mean	Standard deviation	Min.	25th	50th	75th	Max.
Total ω-3 (%)	134	4.86	1.24	2.84	3.96	4.60	5.40	8.87
Docosahexaenoic acid—DHA (%)	134	2.98	0.92	1.50	2.27	2.89	3.51	5.63
Eicosapentaenoic acid—EPA (%)	134	0.64	0.36	0.11	0.41	0.55	0.76	2.44
Alpha-linolenic acid—ALA (%)	134	0.24	0.11	0.07	0.17	0.23	0.29	0.79
Docosapentaenoic acid—DPA (%)	134	1.00	0.23	0.43	0.87	0.98	1.12	1.70

After adjusting for covariates, for each 0.29 μg/m^3^ increase in black carbon concentration lagged 24 h was associated with a 2.6% increase in fibrinogen levels (95% CI = 0.7%, 4.4%) in the LOW total ω-3 tertile and a 2.8% increase (95% CI = 1.3, 4.2) in the MEDIUM ω-3 tertile. However, there was essentially no change in fibrinogen in the HIGH ω-3 tertile (0.2% increase; 95% CI = − 1.7%, 2.1%) (Table 4). Since effect estimates appeared similar for the LOW and MEDIUM tertiles, but different for the HIGH tertile for the main pollutants PM_2.5_ and BC, we combined the LOW and MEDIUM into one group (LOWMED), with all further analyses based on two groupings (HIGH and LOWMED).

**Table 4:** Percent change in biomarkers associated with IQR increases in lagged pollutant concentrations within each tertile of omega-3 fatty acid

Pollutant	Lag hours	*n*	IQR	Tertile	% change (95% CI)	*P* value (individual interactions)	*P* value (overall interaction)
AMP	0–11	125	452	LOW	2.0 (0.6, 3.4)	0.97	0.71
				MED	3.0 (0.9, 5.0)	0.50	
				HIGH	2.0 (− 0.1, 4.1)	Ref.	
Black carbon	0–23	125	0.29	LOW	2.6 (0.7, 4.4)	0.08	0.09
				MED	2.8 (1.3, 4.2)	0.04	
				HIGH	0.2 (− 1.7, 2.1)	Ref.	
Delta-C	0–11	125	0.13	LOW	0.7 (0, 1.4)	0.65	0.88
				MED	0.9 (0, 1.8)	0.97	
				HIGH	0.9 (− 0.9, 2.8)	Ref.	
PM_2.5_	0	117	5.6	LOW	3.7 (1.1, 6.4)	0.11	0.26
				MED	2.7 (0.6, 4.8)	0.26	
				HIGH	0.9 (− 1.4, 3.3)	Ref.	
UFP	0–47	125	1595	LOW	3.7 (1.4, 6.0)	0.43	0.68
				MED	2.6 (0.9, 4.4)	0.93	
				HIGH	2.5 (0.5, 4.5)	Ref.	

Using these two groups, each 5.6 μg/m^3^ increase in PM_2.5_ concentration in the prior 1 h in subjects in the LOWMED ω-3 fatty acid group was associated with a 3.1% increase in fibrinogen levels (95% CI = 1.5%, 4.7%), but only a 0.9% increase (95% CI = − 1.5%, 3.2) in those with HIGH ω-3 fatty acid levels (Table 5). Similarly, each 0.29 μg/m^3^ increase in black carbon concentration lagged 24 h was associated with a 2.7% increase in fibrinogen levels (95% CI = 1.5%, 3.8%) in the LOWMED ω-3 group. However, there was essentially no change in fibrinogen in the HIGH ω-3 group (0.2% increase; 95% CI = − 1.7%, 2.1%). However, there did not appear to be effect modification of the associations between fibrinogen and AMP, UFP, or Delta-C, as there was little difference in effect estimates between the HIGH and LOWMED ω-3 groups (Table 5).

**Table 5:** Percent change in biomarkers associated with IQR increases in lagged pollutant concentrations within each tertile of ω-3 fatty acid

Pollutant	Lag hours	*n*	IQR (μg/m^3^)	Tertile	% change (95% CI)	*P* value	*P* value of interaction
AMP	0–11	125	452	LOWMED	2.3 (1.1, 3.5)	< 0.001	0.77
				HIGH	2.0 (− 0.1, 4.0)	0.06	
Black carbon	0–23	125	0.29	LOWMED	2.7 (1.5, 3.8)	< 0.001	0.03
				HIGH	0.2 (− 1.7, 2.1)	0.83	
Delta-C	0–11	125	0.13	LOWMED	0.8 (0.2, 1.3)	0.01	0.84
				HIGH	0.9 (− 0.9, 2.6)	0.30	
PM_2.5_	0	117	5.6	LOWMED	3.1 (1.5, 4.7)	< 0.001	0.12
				HIGH	0.9 (− 1.5, 3.2)	0.45	
UFP	0–47	125	1595	LOWMED	3.0 (1.5, 4.4)	< 0.001	0.69
				HIGH	2.5 (0.5, 4.5)	0.02	

**Fig. 1:**
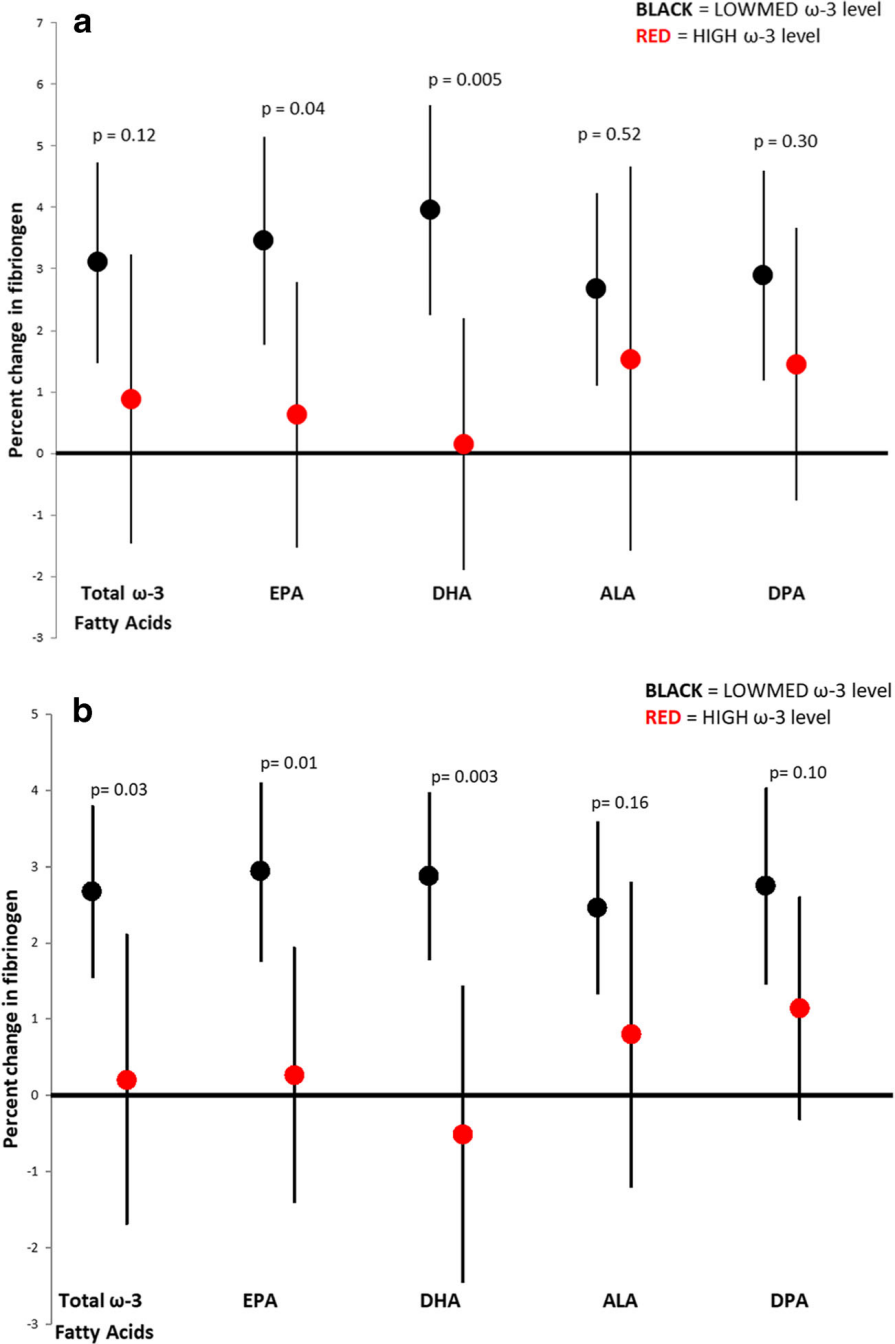
Percent change in fibrinogen associated with each IQR increase in **a** PM_2.5_ concentration in the previous hour and **b** black carbon (BC) concentration in the previous 24 h, by ω-3 fatty acid type and level

Next, we examined whether specific ω-3 fatty acid subgroups (i.e., EPA, DHA, DPA, and ALA) each individually modified the fibrinogen/PM associations. Each 5.6 μg/m^3^ increase in PM_2.5_ concentration in the previous hour was associated with a 3.96% increase in fibrinogen (95% CI = 2.25%, 5.67%) within the LOWMED DHA group, but essentially no change in the HIGH DHA group (0.15%; 95% CI = − 1.89%, 2.2%) (Table 6 and Fig. 1a). This same pattern of effect modification of fibrinogen was also observed for BC (Table 6 and Fig. 1b). For both PM_2.5_ and BC, we observed a similar pattern of effect modification of the fibrinogen/pollutant association by EPA and DHA, but not ALA or DPA (Table 6, Fig. 1a, b). Last, we evaluated whether the same effects were noted for all lag times. We observed similar patterns of effect modification of the fibrinogen/PM_2.5_ 1 h association and the fibrinogen/black carbon 24 h association for other PM_2.5_ and BC averaging/lag times (Table S2 and S3).

**Table 6:** Percent change in fibrinogen associated with each 5.6 μg/m^3^ increase in PM_2.5_ concentration in the previous 1 h, and BC in the previous 24 h within each type of ω-3 fatty acid

Pollutant	ω-3 fatty acid	Level	% change (95% CI)	*P* values	*P* value of interaction
PM_2.5_	EPA	LOWMED	3.4 (1.8, 5.1)	< 0.001	0.04
		HIGH	0.6 (− 1.5, 2.8)	0.57	
	DHA	LOWMED	4.0 (2.3, 5.7)	< 0.001	0.005
		HIGH	0.2 (− 1.9, 2.2)	0.88	
	ALA	LOWMED	2.7 (1.1, 4.2)	0.001	0.52
		HIGH	1.5 (− 1.6, 4.7)	0.33	
	DPA	LOWMED	2.9 (1.2, 4.6)	0.001	0.30
		HIGH	1.5 (− 0.8, 3.7)	0.2	
Black carbon	EPA	LOWMED	2.9 (1.8, 4.1)	< 0.001	0.01
		HIGH	0.3 (− 1.4, 1.9)	0.75	
	DHA	LOWMED	2.9 (1.8, 4.0)	< 0.001	0.003
		HIGH	− 0.5 (− 2.4, 1.4)	0.6	
	ALA	LOWMED	2.5 (1.3, 3.6)	< 0.001	0.16
		HIGH	0.8 (− 1.2, 2.8)	0.43	
	DPA	LOWMED	2.7 (1.5, 4.0)	< 0.001	0.10
		HIGH	1.1 (− 0.3, 2.6)	0.12

## Discussion

In patients with active coronary disease (i.e., those experiencing a myocardial infarction or those treated for stable ischemic heart disease in our cardiac catheterization laboratory), we previously found that increases in fibrinogen were associated with increased concentrations of PM air pollution (PM_2.5_, BC, UFP, and AMP) in the previous 1 to 48 h (Croft et al. 2017).

However, as hypothesized, we observed that increased fibrinogen was associated with increased PM_2.5_ and black carbon concentrations only in those with LOW/MEDIUM ω-3 fatty acid levels, but there was no effect of either pollutant on fibrinogen when serum ω-3 fatty acid levels were HIGH. Further, this same pattern of effect modification by ω-3 fatty acids was seen across multiple lag times (i.e., lag hours 0, 0–11, 0–23, 0–47, 0–71, and 0–95) for both PM_2.5_ and BC. Consistent with our hypothesis, this same pattern was observed for two ω-3 fatty acids found in fish oil (EPA and DHA), but not an ω-3 fatty acid derived from plants (ALA).

Previous studies have reported that fish oil ω-3 fatty acid supplements blunted or lessened changes in markers of autonomic dysfunction associated with short-term increases in ambient PM concentrations, including successive normal RR intervals differing by more than 50 ms (pNN50), the root mean square of the sum of the square of differences between adjacent intervals (r-MSSD), and the standard deviation of normal to normal intervals (SDNN) (Romieu et al. 2005; Tong et al. 2012). A recent study reported an attenuation of fibrinogen levels after exposure to concentrated ambient air pollution particles in subjects who underwent 4 months of fish oil supplementation prior to exposure (Tong et al. 2015). Our finding that elevated plasma levels of fibrinogen associated with acutely increased PM concentrations were attenuated by high levels of total ω-3 fatty acids and specifically fish oil-derived EPA and DHA is consistent with these studies. Furthermore, our study results are strengthened by our finding of consistent effect modification by EPA and DHA for multiple pollutants (PM_2.5_ and BC) at all lag times studied (1–96 h) (Tables S2 and S3). These findings provide additional evidence for fish oil’s mitigation of adverse changes in fibrinogen with an important strength being the measurement of the actual blood levels of specific ω-3 fatty acid components.

Other studies not focused on air pollution have examined whether ω-3 fatty acid supplements reduced plasma fibrinogen concentrations. Lee et al. (2006) reported no change in overall fibrinogen level following 3 months’ treatment with 1 g/day of OMACOR® ω-3 fatty acids, and no change in fibrinogen binding following supplementation with OMACOR® (850–882 mg EPA and DHA ω-3 fatty acids) for 6 weeks (Mackay et al. 2012). Similar to our study, Mackay et al. (2012) also used plasma levels of ω-3 fatty acids, while Lee et al. (2006) relied on supplementation history. Our measuring of blood plasma levels of fatty acids is preferable to relying on dietary or supplement information when studying cardiovascular disease outcomes (Burr et al. 1989; Mozaffarian and Wu 2011), as the high variability in absorption makes extrapolating blood levels from survey-based methods less reliable (Superko et al. 2013).

Blunting or lessening of PM/fibrinogen associations by EPA and DHA may be related to their anti-inflammatory properties (Chapkin et al. 2009). The general mechanism of EPA and DHA’s anti-inflammatory effect likely involves the attenuation of cyclooxygenase (COX) activity, which normally promotes thromboxane production, perpetuating inflammation and thrombosis (Im 2016). More specifically, EPA and DHA produce resolvins which act via inhibition of the interleukin IL-1β while DHA produces two additional anti-inflammatory specialized pro-resolving mediators, protectins, and maresins (Calder 2015). Protectins inhibit tumor necrosis factor alpha (TNF-α) and interleukin-1-beta (IL-1β) production (Calder 2015) and maresins inhibit leukotriene A4 hydrolase (LTA4H) (Serhan et al. 2015). Though ALA is associated with reductions in some of the similar anti-inflammatory cells as DHA and EPA (i.e., IL-6), the plant-based ALA and fish oil-based DHA and EPA are suspected to act by independent mechanistic pathways (Anderson and Ma 2009). Also, though our study did not show effect modification of DPA on the adverse changes in fibrinogen, there is a growing literature on DPA’s (intermediary ω-3 fatty acid between EPA and DHA) cardiovascular benefit, thought to be through anti-inflammatory pathways and beneficial lipid and platelet effects (Byelashov et al. 2015). Thus, it is possible that our finding of effect modification of PM-mediated inflammation by DHA and EPA, but not DPA or the predominantly plant-based ALA, is due to modification of an inflammatory pathway unique to EPA and DHA. Elucidating the respective mechanisms of fish and plant-based ω-3 fatty acids on PM-mediated inflammation is an important area of future research.

It is unclear why ω-3 fatty acids modified associations between fibrinogen and PM_2.5_ and BC, but not fibrinogen associations with UFP, AMP, and Delta-C. Whether a unique inflammatory pathway in response to PM_2.5_ and BC is particularly susceptible to effect modification by omega-3 fatty acids has not been studied. Future studies using larger populations and more spatially resolved pollutant concentrations (e.g., land use regression estimated concentrations at each subject’s residence) or even personal measurements may provide a clearer understanding of this issue.

Although our plasma measurements of ω-3 fatty acids at the same time as our fibrinogen biomarker measurements and matched pollution data were strengths of this study, there were also several limitations. First, our limited sample size (*n* = 135) likely resulted in reduced statistical power. However, inference on effect modification of the PM/fibrinogen association by ω-3 fatty acids was primarily made by considering whether there were similar patterns of effect modification across multiple pollutants (e.g., PM_2.5_ and BC), multiple lag times (lag hours 0, 0–11, 0–23, 0–47, 0–71, 0–96), and multiple ω-3 fatty acid subgroups (e.g., EPA, DHA, ALA), and not on whether one individual test was statistically significant. Second, we used ambient air pollution data from a central monitoring station for each subject without the ability to account for the distance of their home or work from the monitoring site, likely resulting in non-differential exposure misclassification and underestimates of effects. Third, we were unable to control for differences in socioeconomic status or medication history/usage between subjects, potentially resulting in residual confounding of our comparisons of the fibrinogen/PM associations between subjects in the HIGH and LOWMED ω-3 groups. Fourth, though our use of laboratory measurement of plasma ω-3 levels (and specifically EPA and DHA) provides an accurate assessment of ω-3 levels, we did not have data on regular dietary fish intake or fish oil supplementation history. Without documentation of each subject’s regular ω-3 intake, we cannot determine whether the observed beneficial fish oil-derived ω-3 effect was due to fish oil supplementation, dietary fish intake, or both (Mohebi-Nejad and Bikdeli 2014).

## Conclusions

Increased circulating ω-3 fatty acid concentrations appeared to blunt the adverse changes in fibrinogen associated with increased PM_2.5_ and black carbon concentrations, but not other pollutants. Furthermore, this protective effect appeared to be driven by fish oil-derived ω-3 fatty acids (i.e., EPA and DHA), but not a plant-derived ω-3 fatty acid (ALA). The mechanism(s) of ω-3 fatty acid’s effect modification of the adverse effects of air pollution on fibrinogen, and the potential for regular ω-3 intake or supplementation to block adverse inflammatory effects of air pollution in populations regularly exposed to air pollution deserves further study.

## Electronic supplementary material

ESM 1 (DOCX 34 kb)
