# Supplementary material for: Do elevated blood levels of omega-3 fatty acids modify effects of particulate air pollutants on fibrinogen?
Source: Air Qual Atmos Health. 2018 Jun 3;11(7):791–9. doi: 10.1007/s11869-018-0586-0 (PMC6097058; doi:10.1007/s11869-018-0586-0)
Supplement: Supplementary file 1 — (DOCX 34 kb) [file 11869_2018_586_MOESM1_ESM.docx]

**Supplemental Material**

**Do elevated blood levels of omega-3 fatty acids modify effects**

**of particulate air pollutants on fibrinogen?**

Daniel Croft MD, MPH,^1^ Robert Block, MD, MPH^2^ Scott J. Cameron MD, PhD,^2^

Kristin Evans PhD,^3^ Charles J. Lowenstein, MD,^2^ Frederick Ling, MD,^2^

Wojciech Zareba, MD, PhD,^2^ Philip K. Hopke, PhD,^3,4^ Mark J. Utell, MD,^1,5^

Sally W. Thurston PhD,^6^ Kelly Thevenet-Morrison, MS,^3^ David Q. Rich, ScD ^1,3,5^

1. Division of Pulmonary and Critical Care, Department of Medicine, University of Rochester Medical Center, Rochester, NY.
2. Division of Cardiology, Department of Medicine, University of Rochester Medical Center, Rochester, NY.
3. Department of Public Health Sciences, University of Rochester Medical Center, Rochester, New York
4. Institute for a Sustainable Environment, and Center for Air Resources Engineering and Science, Clarkson University, Potsdam, New York
5. Department of Environmental Medicine, University of Rochester Medical Center, Rochester, New York
6. Department of Biostatistics and Computational Biology, University of Rochester, Rochester, New York

All Authors and Institutions are in the United States of America.

**Corresponding author:** Daniel P. Croft MD, MPH

University of Rochester Medical Center

Pulmonary and Critical Care Division

601 Elmwood Avenue Box 692

Rochester, NY 14642

Phone: 585 275 4161

Fax: 585 271 1171

Email: daniel_croft@urmc.rochester.edu

**Table of Contents:**

Page 3: Supplemental Table S1

Page 4: Supplemental Table S2

Page 5: Supplemental Table S3

**Table S1.** Distribution of the concentrations of PM_2.5_ and BC concentrations, within tertiles of ω-3 concentrations

|  |  |  |  |  |  |  |  |  |
| --- | --- | --- | --- | --- | --- | --- | --- | --- |
| **Pollutant** | **ω-3 Tertile** | **N** | **Mean** | **Standard Deviation** | **25^th^** | **50^th^** | **75^th^** | **IQR** |
| **PM_2.5_**  **(µg/m^3^)** | LOW | 40 | 6.6 | 3.1 | 4.3 | 6.1 | 7.3 | 3 |
|  | MEDIUM | 43 | 6.8 | 3.8 | 3.7 | 5.4 | 10.1 | 6.4 |
|  | HIGH | 43 | 6.8 | 3.3 | 4.2 | 6.4 | 9 | 4.8 |
| **Black Carbon (µg/m^3^)** | LOW | 44 | 0.37 | 0.22 | 0.21 | 0.30 | 0.49 | 0.28 |
|  | MEDIUM | 46 | 0.39 | 0.28 | 0.20 | 0.32 | 0.52 | 0.32 |
|  | HIGH | 44 | 0.34 | 0.20 | 0.19 | 0.28 | 0.49 | 0.30 |

**Table S2.** Percent change in fibrinogen associated with each IQR increase in PM_2.5_ concentration at multiple lag times, within each tertile of ω-3 fatty acids

| **Lag hours** | **N** | **IQR** | **Level of ω-3 fatty acids** | **% change**  **(95% CI)** | **P value** | **P value of**  **interaction** |
| --- | --- | --- | --- | --- | --- | --- |
| 0 | 117 | 5.60 | LOWMED | 3.1 (1.5,4.7) | <0.001 | 0.12 |
|  |  |  | HIGH | 0.9 (-1.5,3.2) | 0.45 |  |
| 0-11 | 115 | 4.27 | LOWMED | 3.0 (1.6,4.3) | <0.001 | 0.04 |
|  |  |  | HIGH | 0.6 (-1.3,2.5) | 0.51 |  |
| 0-23 | 117 | 4.00 | LOWMED | 2.9 (1.5,4.2) | <0.001 | 0.009 |
|  |  |  | HIGH | 0.05 (-1.6,1.7) | 0.95 |  |
| 0-47 | 115 | 4.15 | LOWMED | 2.6 (1.2,4.1) | <0.001 | 0.05 |
|  |  |  | HIGH | 0.3 (-1.6,2.2) | 0.74 |  |
| 0-71 | 116 | 4.33 | LOWMED | 2.9 (1.3,4.5) | 0.001 | 0.07 |
|  |  |  | HIGH | 0.3 (-2.0,2.7) | 0.77 |  |
| 0-95 | 112 | 4.10 | LOWMED | 3.0 (1.3,4.7) | 0.001 | 0.03 |
|  |  |  | HIGH | -0.3 (-3.0,2.4) | 0.84 |  |

**Table S3**. Percent change in fibrinogen associated with each IQR increase in Black Carbon (BC) concentration at multiple lag times, within each tertile of ω-3 fatty acids

| **Lag hours** | **N** | **IQR** | **Level of ω-3 fatty acids** | **% change**  **(95% CI)** | **P value** | **P value of interaction** |
| --- | --- | --- | --- | --- | --- | --- |
| 0 | 125 | 0.23 | LOWMED | 0.9 (0.2,1.7) | 0.02 | 0.06 |
|  |  |  | HIGH | -0.1 (-0.8,0.7) | 0.88 |  |
| 0-11 | 125 | 0.33 | LOWMED | 2.3 (1.4,3.3) | <0.001 | 0.10 |
|  |  |  | HIGH | 0.6 (-1.1,2.4) | 0.46 |  |
| 0-23 | 125 | 0.29 | LOWMED | 2.7 (1.5,3.8) | <0.001 | 0.03 |
|  |  |  | HIGH | 0.2 (-1.7,2.1) | 0.83 |  |
| 0-47 | 125 | 0.26 | LOWMED | 2.3 (1.1,3.6) | <0.001 | 0.06 |
|  |  |  | HIGH | 0.1 (-1.8,2.1) | 0.91 |  |
| 0-71 | 125 | 0.20 | LOWMED | 2.0 (0.84,3.1) | 0.001 | 0.02 |
|  |  |  | HIGH | -0.4 (-2.1,1.3) | 0.66 |  |
| 0-95 | 125 | 0.19 | LOWMED | 1.9 (0.6,3.19) | 0.004 | 0.04 |
|  |  |  | HIGH | -0.53 (-2.55,1.49) | 0.61 |  |
